# Resistant hypertension caused by stenosis of the aorta in elderly women: three case reports

**DOI:** 10.1186/s40885-014-0005-2

**Published:** 2014-11-18

**Authors:** Hee Dong Kim, Mi-Na Kim, Su-A Kim, Sue In Choi, Jah Yeon Choi, Ji Hye Seo, Sung Hoon Park, Seong-Mi Park, Wan-Joo Shim

**Affiliations:** Division of Cardiology, Department of Internal Medicine, Korea University Anam Hospital, Korea University College of Medicine, Seoul, 136-705 Korea; Korea University Cardiovascular Center, Korea University Anam Hospital, 73 Inchon-ro, Seoul, 136-705 Seongbuk-gu Korea

**Keywords:** Resistant hypertension, Takayasu arteritis, Aortic stenosis

## Abstract

Arterial hypertension is the most common cardiovascular disease in older than middle-aged patients. Reported incidence of secondary hypertension in this age group is around 10%. To evaluate secondary hypertension, history taking and physical examination should be precise, which are usually not routinely performed in busy outpatient clinic. Here, we report three cases of elderly women who are taking antihypertensive and visited outpatient clinic for uncontrolled hypertension. Their diagnosis of aortic stenosis was delayed, and its presence was suggested by detection of high abdominal aortic flow velocity during transthoracic echocardiography. This case report gives us lessons that suspicion of secondary hypertension and careful physical examination are most important to make diagnosis early.

**Electronic supplementary material:**

The online version of this article (doi:10.1186/s40885-014-0005-2) contains supplementary material, which is available to authorized users.

## Background

Arterial hypertension is the most common cardiovascular disease in older than middle-aged patients. The prevalence of resistant hypertension is around 10% of all hypertensive population [1],[2], and clinicians should always consider possible secondary causes of hypertension in this clinical setting, especially in young adults. Hypertension caused by vascular origin is usually caused by renal artery stenosis and infrequently aortic coarctation. As for stenosis of the aorta, coarctation of the thoracic aorta at the level of the ligamentum arteriosum is considered as congenital vascular lesion. Infrequently, stenosis in distal thoracic or abdominal aorta may cause hypertension, and the pathophysiology of this vascular lesion is considered as vasculitis such as Takayasu arteritis. We report secondary hypertension caused by stenosis of the aorta, probably due to Takayasu arteritis, in three female patients in their early sixties. Diagnosis of aortic stenosis was delayed, and its presence was suggested by detection of high abdominal aortic flow velocity during transthoracic echocardiography.

## Case presentation

### Case 1

**Figure 1:**
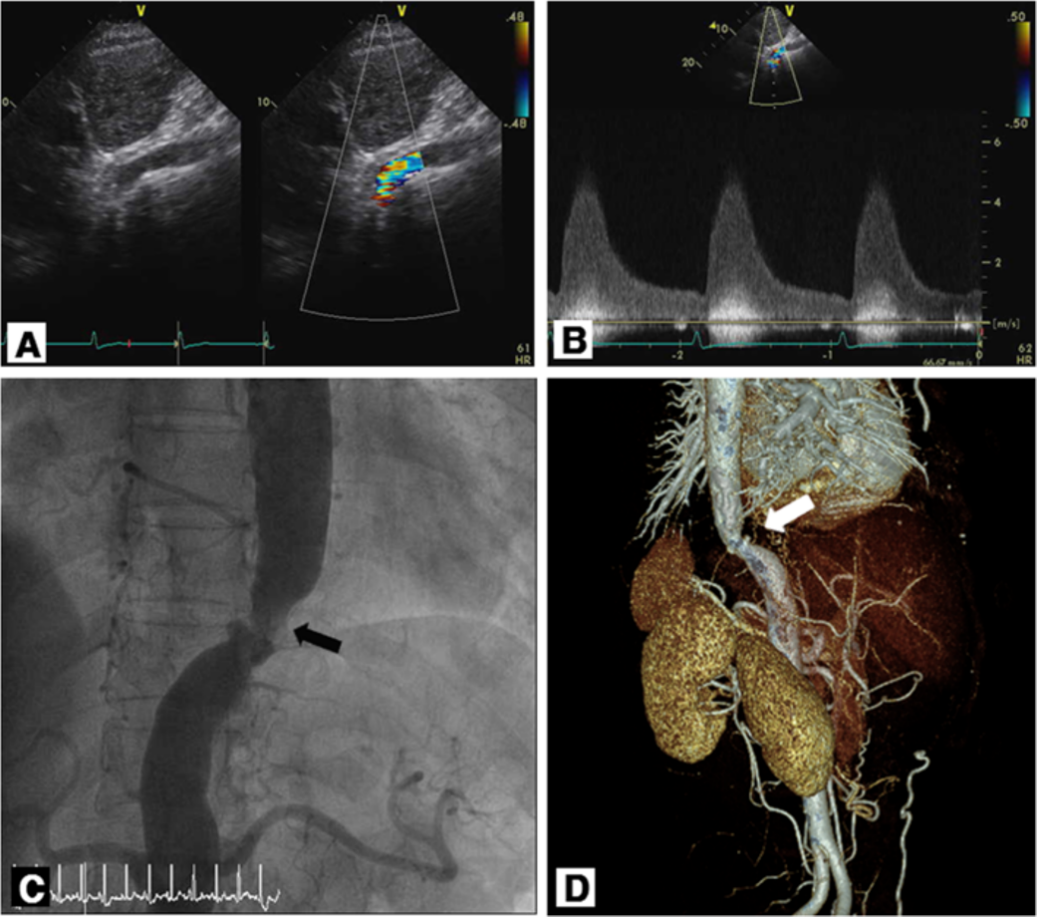
**Evidences of abdominal aorta stenosis in case 1. (A)** Two-dimensional and color Doppler echocardiographic image revealed the stenosis of the descending abdominal aorta (left) and turbulent flow through narrowed segment on color Doppler (right). **(B)** Peak velocity and peak pressure gradient were 5.1 m/s and 105 mmHg, respectively, on continuous wave Doppler echocardiographic image. **(C)** Angiography and **(D)** three-dimensional reconstructed image of computed tomography angiogram revealed a significant stenosis of the suprarenal abdominal aorta (black and white arrows).

A 64-year-old woman presented to the emergency room for muscular pain and headache after a slip down the stairs. Her initial blood pressure was 240/100 mmHg, but it decreased to 200/100 mmHg after resting. She had a history of hypertension for 13 years, and her blood pressure had become less responsive to medication, including calcium channel blocker, beta-blocker, angiotensin receptor blocker, and diuretics. After pain control, she was referred to the department of cardiology for her high blood pressure. For evaluation of uncontrolled hypertension, blood chemistry, Venereal Disease Research Laboratory (VDRL) test, serum renin and aldosterone assay, urinalysis, and electrocardiogram (ECG) were performed. All of these test results were normal except left ventricular hypertrophy (LVH) in ECG. Echocardiogram for LVH was performed and showed increased left ventricular mass (134 g/m^2^) and dilated left atrium (anteroposterior diameter 45.3 mm). The results of the subcostal four-chamber view imaging of the abdominal aorta revealed small-caliber abdominal aorta (Figure 1A) with marked flow acceleration (peak velocity =5.1 m/s) (Figure 1B). At this point, the patient’s blood pressure in the upper and lower extremities was measured. The blood pressure at her right upper extremity was 200/120 mmHg, left upper was 205/110 mmHg, right lower was 100/70 mmHg, and left lower was 110/75 mmHg. Aortic stenosis after branching to left subclavian artery was suspected. Subsequent aortic angiogram (Figure 1C) with aortic pressure measurement showed 88 mmHg systolic pressure gradient at the mid thoracic aorta. The pressure gradient of the aortic arch for both carotid arteries and renal artery was normal. Subsequently, bypass surgery between the ascending aorta and abdominal aorta was carried out successfully. Currently, the patient is taking beta-blockers and diuretics, and her blood pressure is stable without the use of other antihypertensive medication.

### Case 2

A 60-year-old woman was referred to the hospital because of substernal chest pain. She also experienced slowly progressive exertional dyspnea for 3 years. She had a history of hypertension for 10 years with an uncontrolled blood pressure, in spite of administration of multiple antihypertensive medications. Upon admission, her blood pressure was 167/67 mmHg and her heart rate was 68 bpm. Her ECG revealed LVH and following echocardiogram revealed LVH and left atrial enlargement with normal regional wall motion and global systolic function of the left ventricle. However, in subcostal view, the caliber of the abdominal aorta was diminished with increased systolic flow velocity. These findings suggested stenosis of the aorta. Subsequent aorta imaging study revealed stenosed thoracoabdominal aorta. The patient was also found to have multiple stenoses in the coronary arteries and both carotid arteries. During the screening test, ankle brachial index on both legs was decreased (right, 0.53; left, 0.57); the renin level was 19.37 ng/mL/h at supine position, and the VDRL test was not reactive. A diagnosis of Takayasu arteritis was made based on the results of the imaging studies, and other causes of stenosed aorta could be ruled out. Erythrocyte sedimentation rate (ESR) and C-reactive protein (CRP) level were mildly elevated (ESR, 38 mm/h; CRP, 8.19 mg/L), indicating active inflammation, and therefore, a steroid therapy was initiated. Percutaneous intervention for coronary arteries and carotid arteries was performed, and aortic bypass surgery will be considered after stabilization of vascular inflammation.

### Case 3

**Figure 2:**
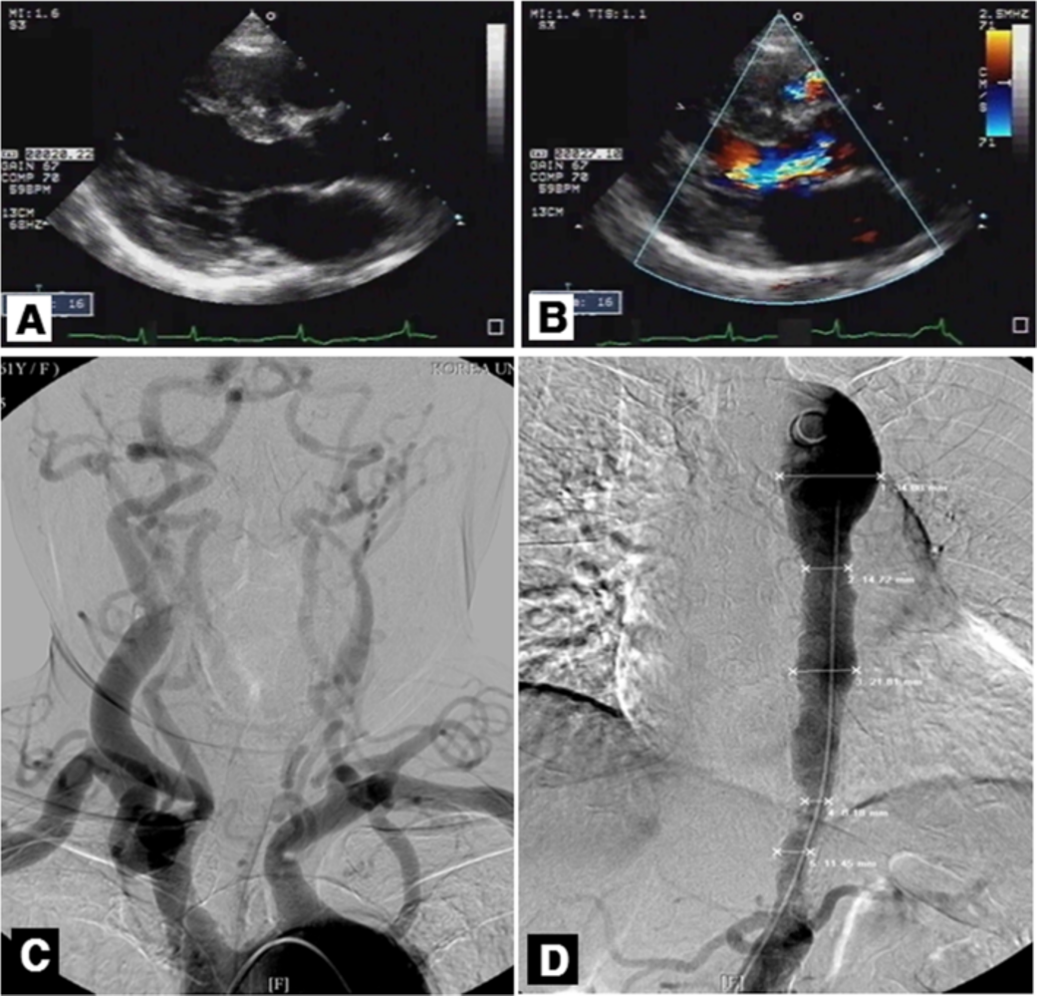
**Echocardiographic and angiographic findings of case 2.** Parasternal long axis view of a two-dimensional echocardiographic image showing **(A)** left ventricular hypertrophy and **(B)** aortic regurgitant jet. Angiography revealed **(C)** total occlusion of the left common carotid artery and **(D)** diffuse narrowing of the thoracoabdominal aorta with a 8.2-mm minimal lumen diameter.

The third case is a 61-year-old woman who presented with cardiac murmur and uncontrolled hypertension despite multiple antihypertensive drugs. At first visit, physical examination revealed high brachial systolic blood pressure of 200/25 mmHg, increased pulse pressure, bounding pulse, and loud to-and-fro murmur at the anterior and back of thorax. Aortic regurgitation was suspected. The results of laboratory test including renin, aldosterone, and VDRL were in normal range. Following echocardiography showed marked LVH and moderate to severe aortic regurgitation (Figure 2A,B). Similar to the case 1 result, the subcostal view revealed small-caliber abdominal aorta with high aortic flow velocity, which suggested aortic stenosis. Subsequent aortogram and carotid angiogram showed complete occlusion of the proximal left common carotid artery and narrowed descending thoracic aorta and proximal portion of the abdominal aorta with normal lower extremity. Aortic bypass surgery with aortic valve replacement was recommended, but the patient refused surgery.

## Conclusions

We described three cases of resistant hypertension caused by stenosis of aorta that was diagnosed in persons who are in their early 60’s. Unfortunately, the detection of aorta stenosis as a cause of hypertension was delayed until early 60’s, and it was incidentally detected by imaging of the abdominal aorta during echocardiographic examination. Takayasu arteritis was diagnosed by typical imaging findings and by exclusion of other causes of stenosis of aorta. Their laboratory findings, which indicated secondary hypertension, especially renin, aldosterone, and VDRL, are unremarkable. The three patients are old and have hypertension; therefore, the atherosclerotic change of the aorta could be possible. But angiographic and ultrasonographic findings of these patients were compatible for Takayasu arteritis, rather than diffuse and multiple atherosclerosis.

Takayasu arteritis is a chronic inflammatory disease, which usually involves the aorta and its main branches [3]. The onset of Takayasu arteritis is usually insidious with nonspecific symptoms such as fever or night sweat and thus remains easily unrecognized until late (sclerotic) stage, but it is usually diagnosed before the patient enters his 50’s [4]. After acute inflammatory period, the development of arterial impairment may cause symptoms that vary according to the involved vasculature. The common complaints of patients with Takayasu arteritis are claudication (upper extremities are involved more often than lower extremities), bruits, and asymmetrical pulses in the right and left extremities. Aneurysm involving ascending aorta can also be a complication of the disease. The development of hypertension in Takayasu arteritis occurs in more than one-half of the patients and is usually a consequence of renal artery stenosis. In rare cases, hypertension can also be induced by suprarenal aortic coarctation.

Suspicion of secondary hypertension caused by vascular stenosis of renal artery or suprarenal aorta usually begins when the examiner notices audible bruits or blood pressure differences between extremities. All the hypertension guidelines recommend measurement of blood pressure in both arms and careful auscultation of the carotid, renal, and heart sounds and murmur [5]. In the cases presented here, systolic bruit was not clearly evident in the first and second cases, and bruit was probably missed due to concomitant loud to-and-fro aortic regurgitation murmur in the third case. However, if at the initial stage, blood pressure was measured in the upper and lower extremities, aortic stenosis as a cause of resistant hypertension could have been detected earlier in our three cases. It is important to note that early diagnosis is critical to prevent vascular complications as the progression of Takayasu arteritis could be stopped by administration of glucocorticoid treatment [6].

Another interesting point in our cases is that aortic stenosis was diagnosed during transthoracic echocardiographic examination. Generally, echocardiographic evaluation for hypertensive patients was helpful to develop a diagnosis and treatment strategy and to estimate a prognosis. Echocardiographic examination provides the information about left ventricular geometry and function, and left atrial volume and function. Therefore, clinicians could evaluate the presence of target organ damage and the changes of cardiac structure and function by resistant or secondary hypertension. During echocardiographic examination, cardiac subcostal four-chamber view showed venous connection to the right atrium and abdominal aorta. The examiner usually concentrates to get good images of cardiac structures and intracardiac blood flow characteristics. In addition, careful abdominal aortic imaging during echocardiographic examination can provide valuable information such as size of aorta, intimal flap, and atherosclerotic plaque along the aorta wall. In all three cases, high velocity jet by Doppler examination inside the abdominal aorta and decreased size of the aorta seen by echocardiographic examination prompted clinicians to search for the stenosis of the middle aorta. Blood pressure measurement in the four extremities after observing high flow velocity in the abdominal aorta could diagnose aorta stenosis immediately.

Our cases had several instructive points. First, even in elderly patients, secondary cause of hypertension needs to be considered when there is uncontrolled blood pressure despite use of multiple drugs. The medical history of the three cases revealed that blood pressure was left uncontrolled for at least 5 years. Second, during echocardiography examination, it is important to get a good image of the abdominal aorta and its flow velocity. Third, a complete and thorough physical examination is always important. Even in presumably primary hypertension cases, an initial examination should be included. Lastly, blood pressure measurement of the extremities should be performed, especially when a patient presents with resistant hypertension.

## Consent

Written informed consent was obtained from the patients for publication of this Case Report and any accompanying images. A copy of the written consent is available for review by the Editor-in-Chief of this journal.

## Authors’ original submitted files for images

Below are the links to the authors’ original submitted files for images. Authors’ original file for figure 1 Authors’ original file for figure 2

## Abbreviations

ECG: electrocardiogram
LVH: left ventricular hypertrophy
VDRL: Venereal Disease Research Laboratory
