# Supplementary figures and images for: Resistant hypertension caused by stenosis of the aorta in elderly women: three case reports
Source: Clin Hypertens. 2014 Nov 18;20:5. doi: 10.1186/s40885-014-0005-2 (PMC4745143; doi:10.1186/s40885-014-0005-2)

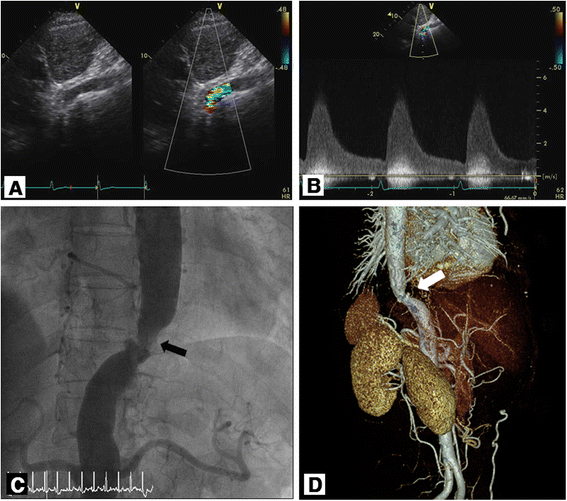

Supplement: Supplementary file 1 — Authors’ original file for figure 1 [file 40885_2014_5_MOESM1_ESM.gif]

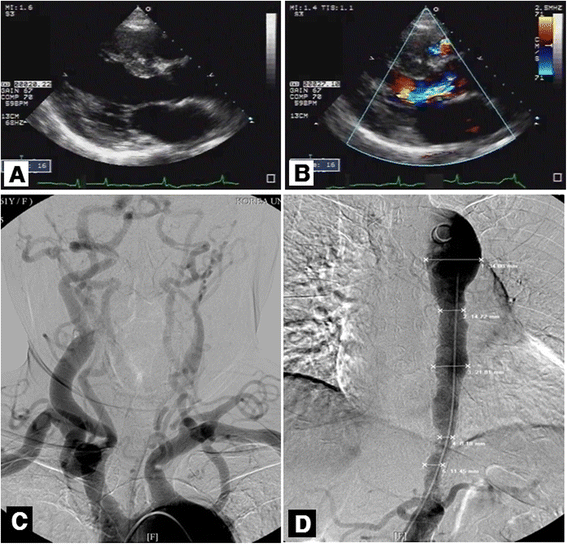

Supplement: Supplementary file 2 — Authors’ original file for figure 2 [file 40885_2014_5_MOESM2_ESM.gif]
